# Arsenic Trioxide Enhances the Radiation Sensitivity of Androgen-Dependent and -Independent Human Prostate Cancer Cells

**DOI:** 10.1371/journal.pone.0031579

**Published:** 2012-02-20

**Authors:** Hui-Wen Chiu, Yi-An Chen, Sheng-Yow Ho, Ying-Jan Wang

**Affiliations:** 1 Department of Environmental and Occupational Health, National Cheng Kung University, Medical College, Tainan, Taiwan; 2 Sinlau Christian Hospital, Tainan, Taiwan; National Cancer Institute, United States of America

## Abstract

Prostate cancer is the most common malignancy in men. In the present study, LNCaP (androgen-sensitive human prostate cancer cells) and PC-3 cells (androgen-independent human prostate cancer cells) were used to investigate the anti-cancer effects of ionizing radiation (IR) combined with arsenic trioxide (ATO) and to determine the underlying mechanisms *in vitro* and *in vivo*. We found that IR combined with ATO increases the therapeutic efficacy compared to individual treatments in LNCaP and PC-3 human prostate cancer cells. In addition, combined treatment showed enhanced reactive oxygen species (ROS) generation compared to treatment with ATO or IR alone in PC-3 cells. Combined treatment induced autophagy and apoptosis in LNCaP cells, and mainly induced autophagy in PC-3 cells. The cell death that was induced by the combined treatment was primarily the result of inhibition of the Akt/mTOR signaling pathways. Furthermore, we found that the combined treatment of cells pre-treated with 3-MA resulted in a significant change in AO-positive cells and cytotoxicity. In an *in vivo* study, the combination treatment had anti-tumor growth effects. These novel findings suggest that combined treatment is a potential therapeutic strategy not only for androgen-dependent prostate cancer but also for androgen-independent prostate cancer.

## Introduction

Prostate cancer represents a major health problem for men worldwide. Previous studies have demonstrated that the activation of androgen receptors (AR) by androgens is required for the growth and survival of prostate cancer cells. Furthermore, most prostate tumors are androgen-dependent in the beginning [1]. However, over time, the tumor recurs in an androgen-refractory manner and present with a more aggressive and metastatic phenotype, which is resistant to further hormonal manipulation [2]. Because androgens play important roles in the growth and survival of prostate cancer cells, growing evidence suggests a significant role for Akt in the development of hormone-independent prostate disease [3], [4], [5]. The inhibition of Akt in prostate cells abrogates HER-2/neu-induced AR signaling and cell survival/growth effects in the absence or presence of androgen [4]. Furthermore, successful progression to an androgen-independent state requires intact PI3K signaling [6]. Thus, the inhibition of the Akt pathway is emerging as an attractive clinical objective for the prevention of hormone-refractory disease.

There is now abundant evidence supporting the benefits of high-dose external beam radiotherapy in patients with clinically localized prostate cancer [7]. However, high-dose radiotherapy causes considerable collateral damage to normal cell populations at the treatment site [8]. Thus, the use of chemical modifiers as radiosensitizers in combination with low-dose irradiation may increase the overall therapeutic efficacy. Arsenic has long been used as anticancer agent in traditional Chinese medicine [9]. Recently arsenic trioxide (ATO) has been successfully employed in the treatment of refractory or relapsed acute promyelocytic leukemia (APL), and its efficacy has been confirmed even in patients resistant to conventional chemotherapy [10]. Previous studies have also demonstrated that the combination of ATO and ionizing radiation (IR) is likely to be the most effective strategy for leukemia and solid tumors [11], [12], [13]. ATO could serve as a potent radiation sensitizer and may increase the cure rate of malignant cells. However, the effects and the precise mechanism of combined treatment of ATO and IR against prostate cancer remain unclear.

Autophagy is one of the mechanisms of stress tolerance that maintains cell viability and can lead to tumor dormancy, progression and therapeutic resistance. However, many anticancer drugs could also induce the excessive or prolonged autophagy that triggers tumor cell death. Studies are ongoing to define optimal strategies to modulate autophagy for cancer prevention and therapy and to exploit autophagy as a target for anticancer drug discovery [14]. A number of connections occur upstream of the apoptotic and autophagic machinery, where signaling pathways regulate both processes. Activation of the PI3 kinase/Akt pathway, a well-known method to inhibit apoptosis, also inhibits autophagy [15]. Akt is a serine/threonine protein kinase that plays a critical role in suppressing apoptosis by regulating its downstream pathways [16]. Akt also phosphorylates mammalian target of rapamycin (mTOR), which has been reported to inhibit the induction of autophagy [17]. Both apoptosis and autophagy could be induced in certain tumor cells under the treatment of anti-cancer drugs [18], [19].

Atorvastatin induces autophagy in the androgen receptor negative prostate cancer PC-3 cells through the activation of LC3 transcription [20]. In addition, a recent study has also indicated that a natural BH3 mimetic ((−)-gossypol) induces autophagy in apoptosis-resistant prostate cancer by modulating Bcl-2-Beclin1 interaction at the endoplasmic reticulum [21]. Androgen-independent prostate cancer cells with higher levels of Bcl-2 were more resistant to (−)-gossypol induced apoptosis. However, (−)-gossypol induced similar levels of total cell death in both androgen-dependent and -independent cells; it killed androgen cells mainly through apoptosis, but in androgen-independent cells, the mode of cell death was not fully understood [21]. Recently, it was shown that ATO or IR can also induced autophagy but not apoptosis in cancer cells [22], [23].

In the present study, LNCaP (androgen-sensitive human prostate cancer cells) and PC-3 cells (androgen-independent human prostate cancer cells) were used to investigate the anti-cancer effects of IR combined with ATO. The types of cell death induced by IR combined with ATO were examined. We also investigated the possible mechanisms underlying apoptosis or autophagy in LNCaP and PC-3 cells induced by IR and/or ATO.

## Materials and Methods

### Cell culture and drug treatment

The human prostate cancer cell lines LNCaP (ATCC CRL-1740) and PC-3 (ATCC CRL-1435) were obtained from the American Type Culture Collection (ATCC). The cells were cultured in RPMI 1640 medium (Gibco BRL, Grand Island, NY) that was supplemented with antibiotics containing 100 U/ml penicillin, 100 µg/ml streptomycin (Gibco BRL, Grand Island, NY), and 10% fetal bovine serum (HyClone, South Logan, UT, USA).Cells were incubated in a humidified atmosphere containing 5% CO_2_ at 37°C. Exponentially growing cells were detached with 0.05% trypsin-EDTA (Gibco BRL, Grand Island, NY) in RPMI 1640 medium. For exposure to arsenic trioxide (Sigma Chemical Co.), 1 mM fresh stock solutions were prepared before every experiment and filter-sterilized using a 0.2-µm syringe filter. The reagent was added in a concentrated form to the culture medium and mixed gently. The cultures were subsequently incubated for the times indicated in the figures.

### Treatment with irradiation and cell viability assay

IR was performed with 6 MV X-rays using a linear accelerator (Digital M Mevatron Accelerator, Siemens Medical Systems, CA, USA) at a dose rate of 5 Gy/min. An additional 2 cm of tissue-equivalent bolus was placed on the top of a plastic tissue culture flask to ensure electronic equilibrium, and 10 cm of tissue-equivalent material was placed under the flask to obtain full back-scatter. The treated cells were centrifuged and resuspended in 0.1 ml of PBS. Each cell suspension (0.02 ml) was mixed with 0.02 ml of trypan blue solution (0.2% in PBS). After 1 or 2 min, each solution was placed on a hemocytometer, and the blue stained cells were counted as non-intact.

### Clonogenic assay

Cells were irradiated with 2, 4 or 6 Gy. ATO was added to cells at concentrations of 2 or 5 µM. The cells were trypsinized and counted. Known numbers of cells were subsequently replated in 6-cm culture dishes and returned to the incubator to allow for colony development. After seven days, colonies (containing ≥50 cells) were stained with a 0.5% crystal violet solution for 30 min. Plating efficiency (PE) is the ratio of the number of colonies to the number of cells seeded in the nonirradiated group. Calculation of survival fractions (SFs) was performed using the equation: SF = colonies counted/(cells seeded×PE), taking into consideration the individual PE.

### Determination of early apoptosis

Apoptosis was assessed by observing the translocation of phosphatidyl serine to the cell surface, as detected with an Annexin V apoptosis detection kit (Calbiochem, San Diego, CA, USA), according to our previous report [13].

### Measurement of ROS production

Reactive oxygen species (ROS) production was monitored by flow cytometry using 2,7-dichlorodihydrofluorescein diacetate (DCFH-DA), as described previously [24]. After treatment with IR and/or ATO, the cells were incubated with 20 µM of DCFH-DA for 30 min. The cells were harvested, washed once and resuspended in PBS. Fluorescence was monitored using a flow cytometer.

### Supravital cell staining with acridine orange for autophagy detection

Cell staining with acridine orange (Sigma Chemical Co.) was performed according to published procedures [13]. Cells were pre-treated with autophagy inhibitors 3-methyladenine (3-MA) (Sigma Chemical Co.) at a final concentration of 1 mM for 1 hr before IR treatment.

### Electron microscopy

Cells were fixed with a solution containing 2.5% glutaraldehyde plus 2% paraformaldehyde in 0.1 M cacodylate buffer, pH 7.3, for 1 hr. After fixation, the samples were postfixed in 1% OsO_4_ in the same buffer for 30 min. Ultra-thin sections were subsequently observed under a transmission electron microscope (JEOL JEM-1200EX, Japan) at 100 kV.

### Western blot analysis

Total cellular protein lysates were prepared by harvesting cells in protein extraction buffer for 1 hr at 4°C, as described previously [12]. The densities of the bands were quantified with a computer densitometer (AlphaImager™ 2200 System Alpha Innotech Corporation, San Leandro, CA, USA). The expression of GAPDH was used as the protein loading control. The antibodies for detecting Akt, phospho-Akt, phospho-mTOR, phospho-p70S6K, ERK, phospho-ERK, phospho-PDK1, PDK1, JNK, p38, phospho-p38, Beclin 1, Bax and Bcl-2 were obtained from Cell Signaling Technology (Ipswich, MA, USA); anti-GAPDH was obtained from Abcam (Cambridge, MA, USA); LC3, Atg5 and Atg5-12 were obtained from Abgent (San Diego, CA, USA); anti-p62/SQSTM1 antibody was obtained from MBL (Nagoya, Japan); anti-poly (ADP-ribose) polymerase (PARP) antibody was obtained from Millipore (Billerica, MA, USA); mTOR, p70S6K, phospho-JNK, caspase-3 and cleaved-caspase-3 were obtained from Epitomics (Burlingame, CA, USA).

### *In vivo* tumor growth assays using the PC-3 tumor model in nude mice

All experiments on mice were performed according to the guidelines of our institute (the Guide for Care and Use of Laboratory Animals, National Cheng Kung University). The animal use protocol listed below has been reviewed and approved by the Institutional Animal Care and Use Committee (IACUC) (Approval No: 99138). Six- to eight week-old male nude mice (BALB/cAnN.Cg-Foxn1^nu^/CrlNarl) were acquired from the National Laboratory Animal Center (Taiwan). The animals were housed five per cage at 24±2°C and 50%±10% relative humidity and subjected to a 12-h light/12-h dark cycle. The animals were acclimatized for 1 week prior to the start of experiments and fed with a Purina chow diet and water ad libitum. Tumors were induced by subcutaneous (s.c.) injection of PC-3 cells (2×10^6^ cells in 0.1 mL of PBS) at one site of the right flank. Tumors (visualized as small nodules at the sites of injection) appeared ∼7 days after injection, and the animals were randomly distributed into each group. Mice were treated with: (1) vehicle (PBS), (2) 6 mg/kg ATO three times per week for two weeks (on day 0, 2, 4, 7, 9 and 11), (3) a single dose of 6 Gy IR, or (4) a combination treatment consisting of 6 mg/kg ATO three times per week for two weeks started right after a single dose of 6 Gy IR. Mouse body weights were measured once per week and were used as an indicator of systemic toxicity of the treatment. Tumor growth was measured every two days, and the tumor volume was calculated according to the formula shown below [25]:
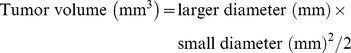
Data are expressed as the relative tumor volume compared to the pretreatment volume measured on day 0. The tumor volume quadrupling time (TVQT, in days) was determined by a best-fit regression analysis. The tumor growth delay (TGD) time is defined as the difference between the TVQT of the treated tumors compared with that of untreated control tumors. The TVQT and TGD time were calculated for each individual mouse and averaged for each group. Tumor growth inhibition rate was calculated as follows: Inhibition (%) = (mean tumor volume of untreated control mice−tumor volume of treated mice)/mean tumor volume of untreated control mice×100.

### Immunohistochemical (IHC) staining analysis

Paraffin-embedded tissue sections (4 µm) were dried, deparaffinized, and rehydrated. After microwave pretreatment in citrate buffer (pH 6.0; for antigen retrieval), the slides were immersed in 3% hydrogen peroxide for 20 min to block the activity of endogenous peroxidase. After intensive washing with PBS, the slides were incubated overnight at 4°C with the LC3 (MBL, Japan), PCNA (ProteinTec Group, Inc., Chicago, USA) and Atg5 (Abgent, San Diego, CA, USA) antibodies. The sections were incubated with a secondary antibody for 1 hr at room temperature, and the slides were developed with the STARR TREK Universal HRP detection kit (Biocare medical, Concord, CA). Finally, the slides were counterstained with hematoxylin. Each slide was scanned at low power (×200).

### Statistical analysis

Data are expressed as the mean ± SD. Statistical significance was determined by using Student's t-test for comparison between the means or one-way analysis of variance with post-hoc Dunnett's test [26]. Differences were considered to be significant when p<0.05.

## Results

### Optimal dose and time selection of IR and ATO for treatment of LNCaP and PC-3 cells

**Figure 1:**
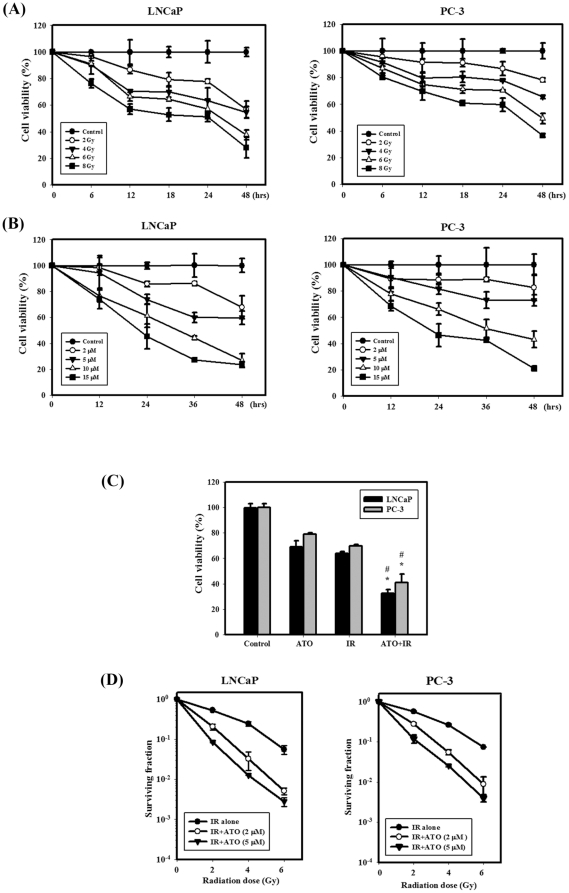
IR dose–response survival curves and cytotoxic effects resulting from ATO and IR in LNCaP and PC-3 cells. (A) Time-course and dose-dependent effects of IR on the viability of LNCaP and PC-3 cells. Cells were treated with 2, 4, 6 or 8 Gy of IR for 6, 12, 18, 24 and 48 hrs. (B) Time-course and concentration-dependent effects of ATO on the viability of LNCaP and PC-3 cells. Cells were treated with 2, 5, 10 or 15 µM of ATO for 12, 24, 36 and 48 hrs. (C) Cytotoxic effects of cells treated with IR (4 Gy) and ATO (5 µM). (D) The radiation dose–response survival curves of LNCaP and PC-3 cells with or without ATO. Data are presented as the mean ± standard deviation from three independent experiments.

The viability of LNCaP and PC-3 cells was observed at different doses of IR (0 to 8 Gy) for 6, 12, 18, 24 and 48 hrs (Fig. 1A). Treatment with IR alone reduced the viability of the LNCaP and PC-3 cells in a dose-dependent manner. Furthermore, the viability of the cells was observed at different concentrations of ATO (0 to 15 µM) for 12, 24, 36 and 48 hrs (Fig. 1B). ATO alone reduced the viability of cells in a concentration-dependent manner. After treatment with 5 µM ATO for 48 hrs, the viability of LNCaP and PC-3 cells was decreased to 60% and 73%, respectively. Figure 1C shows the viability of ATO and IR treated alone or in combination on LNCaP and PC-3 cells. Significantly enhanced toxicity was found for the combination treatment compared with ATO and IR treatment alone in LNCaP and PC-3 cells. Figures 1D shows the radiation dose–response survival curves for LNCaP and PC-3 cells with or without ATO treatment. The survival curves dramatically shifted downward. ATO (2 µM) increased IR-induced clonogenic cell death in LNCaP and PC-3 cells. ATO significantly reduced the survival fraction in a dose-dependent manner compared to IR alone.

### Measurement of apoptosis in LNCaP and PC-3 cells treated with ATO and IR alone or in combination

**Figure 2:**
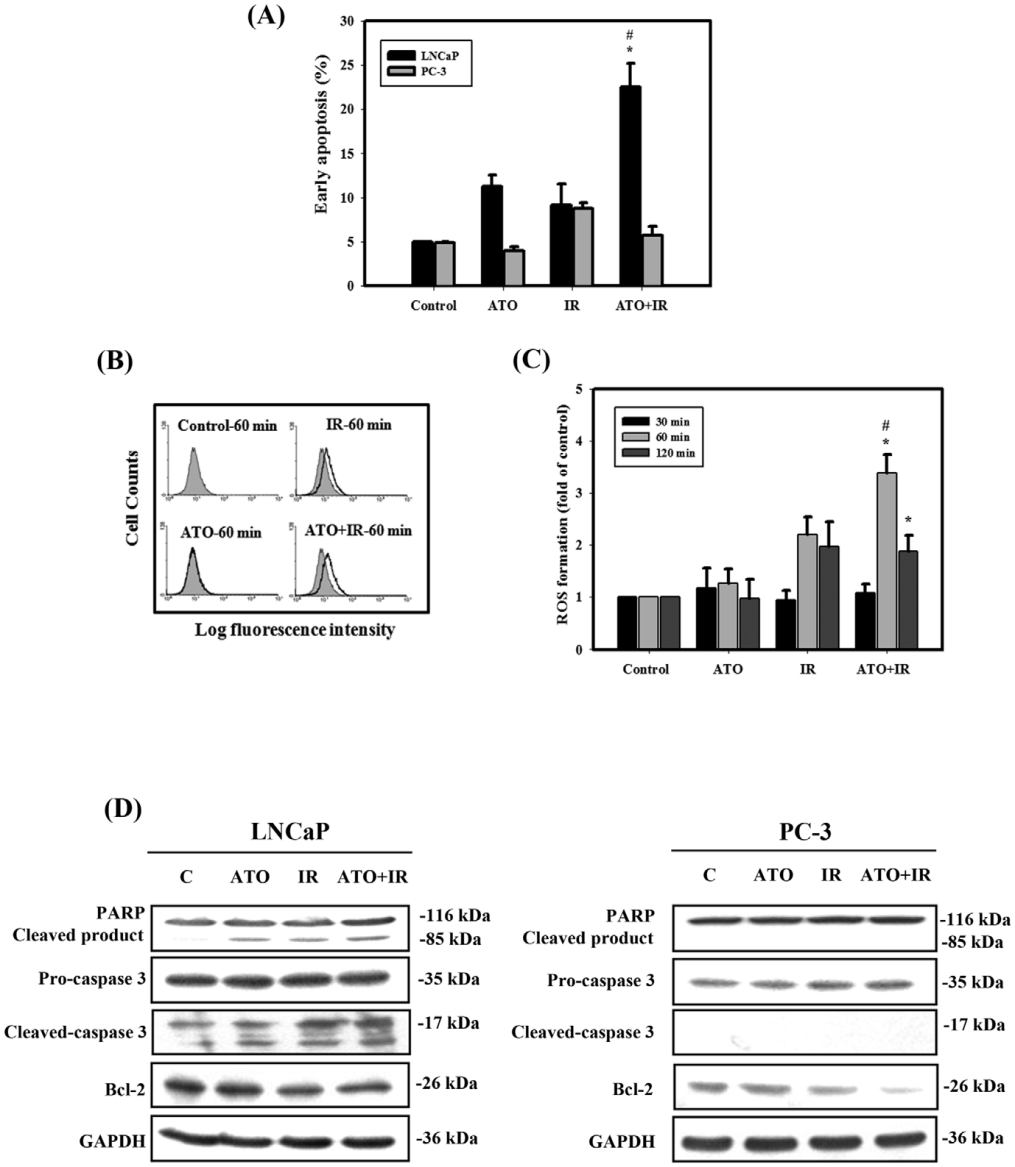
Measurement of apoptosis in LNCaP and PC-3 cells that received various treatments. (A) Early apoptosis detection was measured by flow cytometry with an Annexin V apoptosis detection kit. Cells were treated with IR (4 Gy) and ATO (5 µM) for 48 hrs. #, *p*<0.05, IR versus combined treatment. *, *p*<0.05, ATO versus combined treatment. (B) (C) ROS generation in PC-3 cells treated with 5 µM ATO or 4 Gy IR alone or in combination for 30, 60, 120 min and with DCFH-DA for an additional 30 min. The fluorescence in the cells was immediately assayed using flow cytometry. #, *p*<0.05, IR versus combined treatment. *, *p*<0.05, ATO versus combined treatment. (D) Western blotting of PARP, cleaved-PARP, pro-caspase 3, cleaved-caspase 3and Bcl-2. The level of total GAPDH protein was used as the loading control. Cells were treated with IR (4 Gy) and ATO (5 µM) for 48 hrs. Data are presented as the mean ± standard deviation from three independent experiments.

The induction of apoptotic cell death is a significant mechanism of tumor cells under the influence of radio-/chemotherapy [27]. As shown in Figure 2A, early apoptosis in LNCaP and PC-3 cells was measured by flow cytometry with the Annexin V apoptosis detection kit. Quantitative results showed that the combination treatment induced more apoptotic cell death than treatment alone with ATO or IR in LNCaP cells. By contrast, the occurrence of early apoptosis in PC-3 cells treated with ATO and/or IR was low (less than 10%). ROS generation was further assessed by flow cytometry. An approximately 3.5-fold increase in intracellular peroxide levels was found when PC-3 cells were exposed to the combination treatment for 1 hr (Figs. 2B, 2C). Figure 2D shows the western blot analysis of poly (ADP-ribose) polymerase (PARP), caspase-3, and Bcl-2. The cleavage of PARP by activated caspase-3 results in the formation of an 85-kDa C-terminal fragment. Our results showed that the specific cleavage of PARP and caspase-3 could be found in cells treated with IR, ATO alone or in combination in LNCaP cells. We also analyzed the expression level of Bcl-2, which is a suppressor of programmed cell death, and found that the Bcl-2 proteins declined when treated with IR alone and the combination treatment in both cells.

### Measurement of autophagy in LNCaP and PC-3 cells treated with ATO and IR alone or in combination

**Figure 3:**
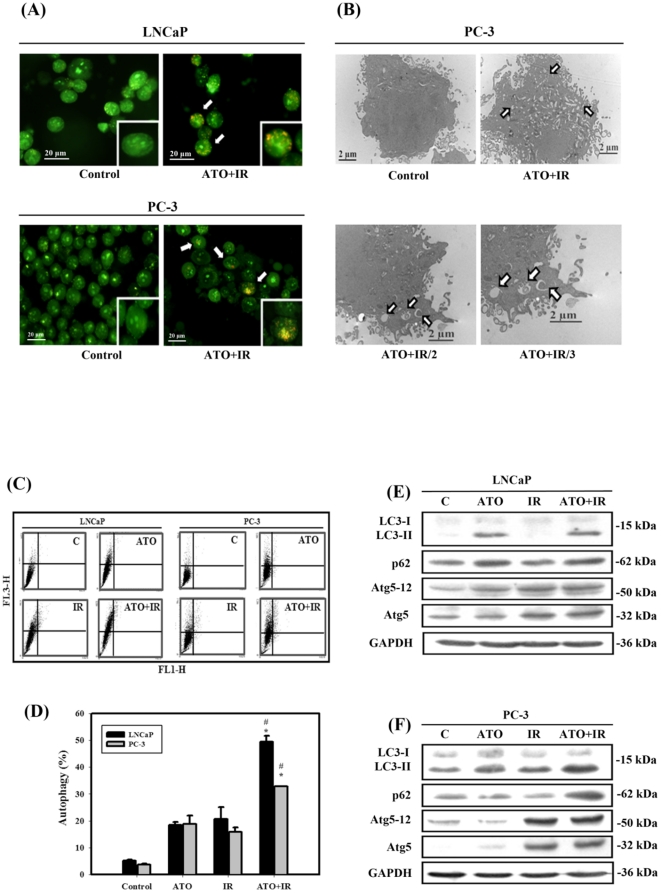
Measurement of autophagy in LNCaP and PC-3 cells that received various treatments. (A) Microphotograph of AVOs in LNCaP and PC-3 cells. Detection of green and red fluorescence in acridine orange (AO)-stained cells was performed using a fluorescence microscope. The *white arrows* point to AVOs. (B) EM microphotographs of PC-3 cells treated with IR (4 Gy) and ATO (5 µM) for 48 hrs. The *white arrows* point to autophagic vacuoles and autolysosomes. (C) Development of AVOs in LNCaP and PC-3 cells. Detection of green and red fluorescence in AO-stained cells using flow cytometry. (D) Quantification of AVOs with AO-stained cells treated with IR (4 Gy) or ATO (5 µM) alone or in combination using flow cytometry. Data are presented as the mean ± standard deviation from three independent experiments. #, *p*<0.05, IR versus combined treatment. *, *p*<0.05, ATO versus combined treatment. (E) (F) Western blotting of LC3-I, LC3-II, p62/SQSTM1, Atg5 and Atg5-12 expression in LNCaP and PC-3 cells. Cells were treated with IR (4 Gy) and ATO (5 µM) for 48 hrs.

Autophagy is characterized by the formation of numerous acidic vesicles, which are called acidic vesicular organelles (AVOs) [23]. Photomicrographs of AVOs were observed via green and red fluorescence in acridine orange (AO)-stained cells with a fluorescence microscope (Fig. 3A). The combined treatment revealed a significant increase in AVOs compared to control groups in LNCaP and PC-3 cells. The ultrastructures of the PC-3 cells for each treatment group were observed by EM photomicrography (Fig. 3B). The combined treatment also resulted in a large number of autophagic vacuoles and autolysosomes in the cytoplasm. In addition, there was no chromatin condensation or nuclear pyknosis, which are characteristic of apoptosis, in any of treated cells. Furthermore, AO staining was quantified using flow cytometry (Figs. 3C, 3D). A significant increase in AO-positive cells was found for cells receiving combined treatment compared to those treated with IR or ATO alone in both cells. To detect the expression of autophagy-related proteins, we performed western blotting with lysates from LNCaP and PC-3 cells receiving each of the different treatments (Fig. 3E, 3F). The expression levels of the LC3-II, p62, Atg5 and Atg5-12 proteins increased with combined treatment in both cells. The combined treatment induced autophagy in LNCaP and PC-3 cells.

### PI3K/Akt signaling pathway is involved in combined treatment-induced cell death and cells undergo both apoptotic and autophagic cell death when exposed to IR and ATO in LNCaP and PC-3 cells

**Figure 4:**
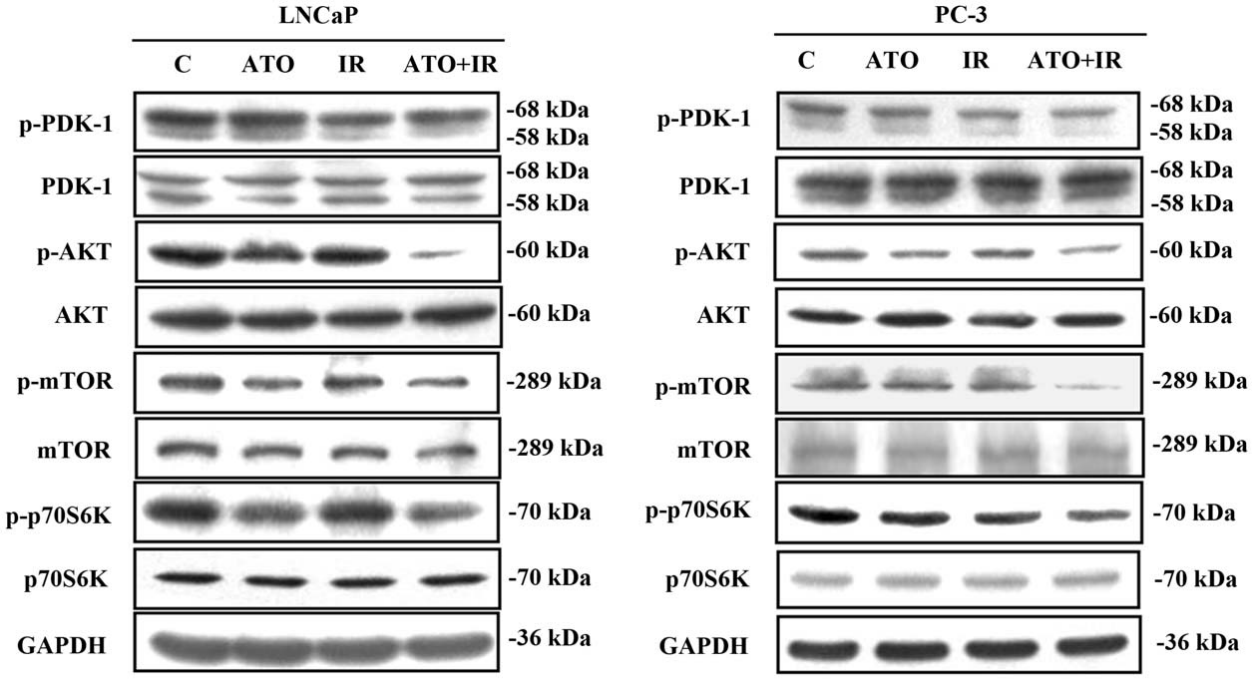
Effects of Akt/mTOR signaling pathway protein expression in LNCaP and PC-3 cells treated with IR and ATO alone or in combination. Cells were treated with IR (4 Gy) and ATO (5 µM) for 48 hrs.

**Figure 5:**
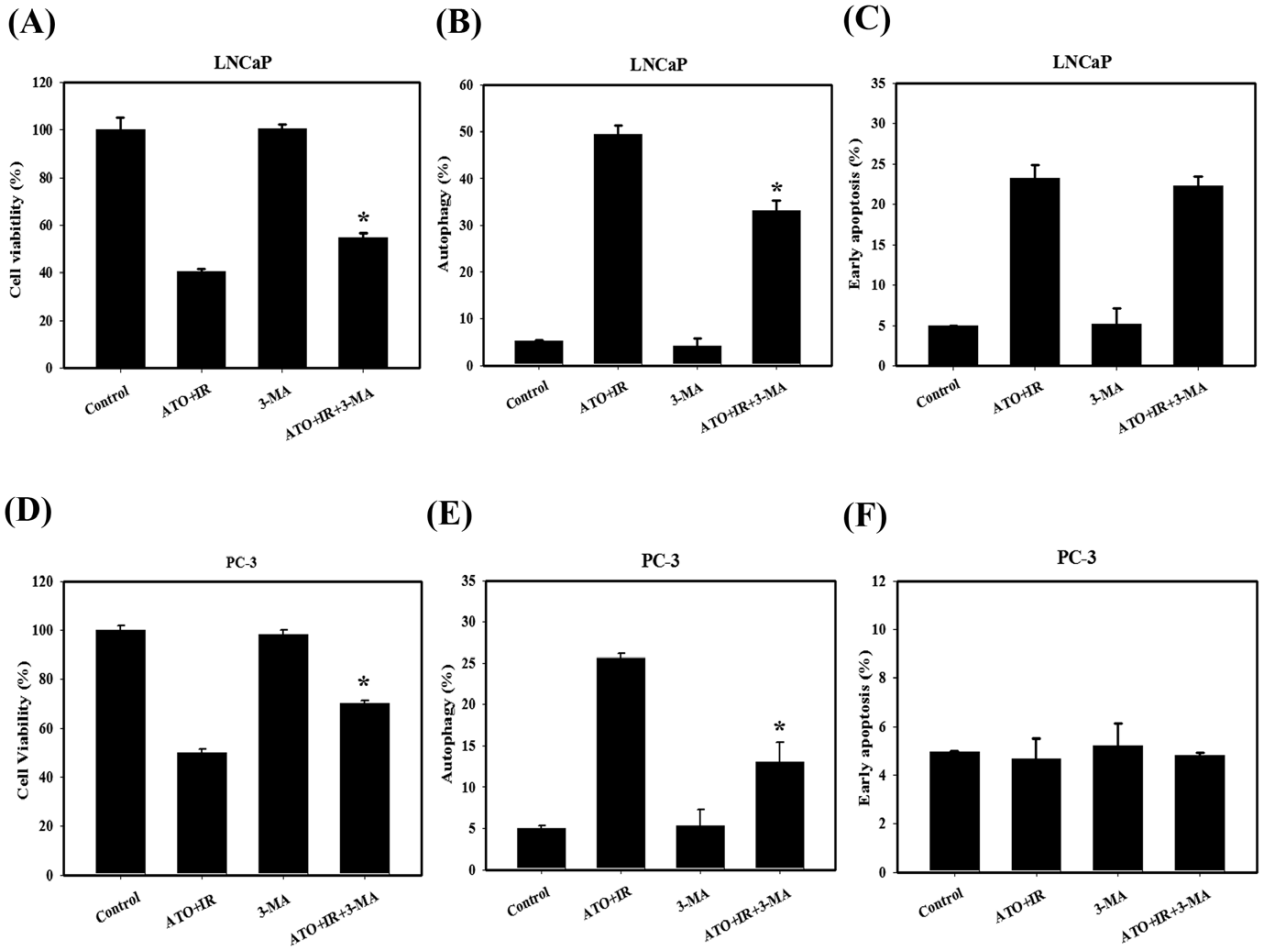
Measurement of autophagy, apoptosis and cytotoxic effects in LNCaP and PC-3 cells pre-treated with 3-MA. (A)(D) Effects of 3-MA on cytotoxicity induced by combined treatment. (B)(E) Early apoptosis was measured by flow cytometry with Annexin V. (C)(F) Quantification of AVOs with AO using flow cytometry. *, *p*<0.05, ATO+IR versus ATO+IR+3-MA.

To investigate whether the PI3K/Akt signaling pathway was involved in combined treatment-induced cell death, we performed western blotting to detect the protein phosphorylation state (Figs. 4A, 4B). Phosphorylated proteins that are related to the PI3K/Akt signaling pathways were also examined. The results show that phosphorylation of Akt, mTOR, p70S6K and PDK1 decreased in cells treated with the combined treatment compared with the control. Next, we investigated whether the inhibition of autophagy could change the percentage of viable cells that had been treated with ATO and IR (Fig. 5). For this purpose, we utilized 3-methyladenine (3-MA) (an autophagy inhibitor). Annexin V and AO staining were quantified using flow cytometry. The combined treatment of LNCaP and PC-3 cells pre-treated with 3-MA showed no effect in apoptotic cells (Fig. 5C, 5F), and a significant decrease in AO-positive cells (Fig. 5B, 5E) compared to combined treatment. Furthermore, we examined whether inhibition of autophagy could alter the cytotoxicity of the combined treatment (Fig. 5A, 5D). We found a significant decrease in cytotoxicity compared with the cells receiving the combined treatment without pre-treatment with 3-MA. These results suggest that ATO combined with IR can induce autophagic cell death in LNCaP and PC-3 cells.

### Tumor growth in nude mice was suppressed by IR and ATO

**Figure 6:**
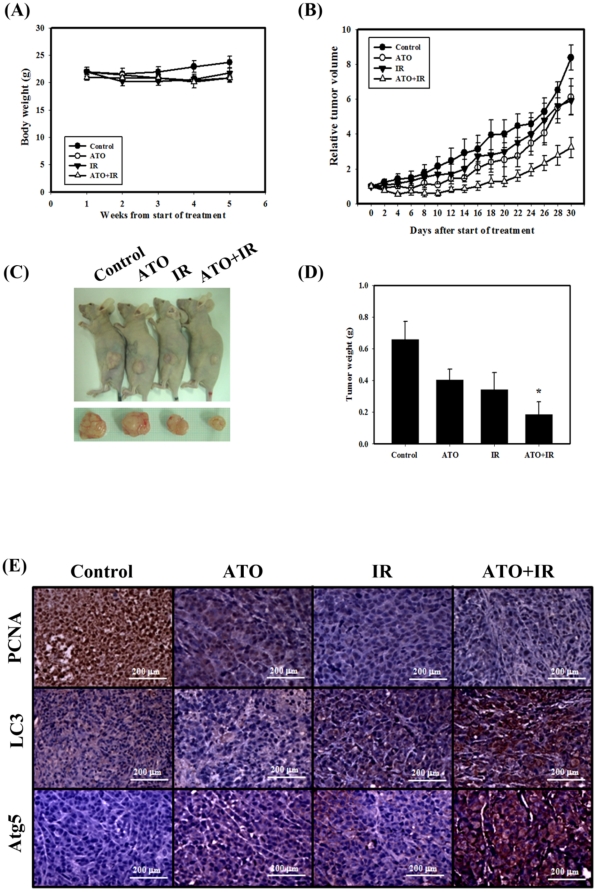
Tumor growth and body weight of tumor-bearing mice treated with IR (6 Gy) or ATO (6 mg/kg×6) alone or in combination. (A) Measurement of body weight in nude mice once per week. (B) Measurement of tumor volume in nude mice every two days. Data are presented as the relative tumor volume (mean ± standard error) normalized to the initial tumor volume measured on day 0. (C) Direct observations of mice with tumors. After the experiments, the mice were sacrificed, and the tumors were removed. (D) Measurement of tumor weight in the nude mice after sacrifice. (E) Immunohistochemical (IHC) staining of PC-3 mouse xenograft tissues. IHC was used to determine the expression levels of PCNA, LC3 and Atg5 (×200 objective magnification).

Next, we next evaluated the anti-tumor growth effect of IR and ATO *in vivo*. Tumors were induced by s.c. injection of PC-3 cells into nude mice. We measured the body weight of the mice every week and the tumor volume every two days. The results of this study demonstrated that none of the treatment regimens produced any loss of body weight, which may be a sign of toxicity (Fig. 6A). The combined treatment suppressed tumor volume and tumor weight in nude mice compared with ATO or IR treatments alone (Figs. 6B, 6C, 6D). As shown in Table 1, the tumor volume quadrupling time (TVQT) of the control group was measured on Day 18 (17.9±2.2). The tumor growth delay (TGD) time for the 18-day treatment of ATO alone was 11 days, which was not significantly different than for the control group (*p* = 0.11). IR alone produced a TGD time of 8.9±5.1 days (*p* = 0.17 compared to the control group). The combination treatment resulted in a TGD time of 39.5±8.3 days (*p*<0.05, compared with IR alone or ATO alone). The combination therapy of IR and ATO resulted in a tumor growth inhibition of 74% on Day 18. Thus, the combination treatment possesses anti-tumor growth effect *in vivo*. Next, the PCNA, LC3 and Atg5 expression pattern in the PC-3 tumors were examined by IHC staining (Fig. 6E). PCNA expression was decreased and LC3 and Atg5 were increased in the combined treatment compared with ATO or IR treatment alone.

**Table 1:** Comparison of tumor growth inhibition, tumor volume quadrupling time, and tumor growth delay time of PC-3 tumors in nude mice.

	Number of mice	Inhibition*(%)	Tumor volume quadrupling time (days)	Tumor growth delay time (days)	p value (t test)#Control ATO IR		
Control	5	-	17.9±2.2	-			
ATO (6 mg/kg×6)	5	53.0±11.4	29.0±5.4	11.1±5.4	0.11		
IR (6 Gy)	5	61.4±11.2	26.8±5.1	8.9±5.1	0.17	0.77	
ATO+IR	5	74.4±9.9	47.4±8.3	39.5±8.3	<0.05	<0.05	<0.05

*Tumor growth inhibition rate was calculated based on the tumor volume on Day 18.

# *p* values for comparison of tumor growth delay time.

## Discussion

Currently, the progression of prostate cancer to a state of androgen independence remains the primary obstacle to improved patient survival. Thus, novel treatment strategies that are useful for androgen-independent disease need to be identified [28]. In the present study, a combination of IR and ATO showed potential as a therapeutic strategy for the treatment of prostate cancer (androgen-dependent and-independent) (Fig. 1). Lian et al. reported that the natural BH3-mimetic (−)-gossypol preferentially induces autophagy in androgen-independent prostate cancer cells that are resistant to apoptosis, whereas apoptosis is preferentially induced in androgen-dependent cells [29]. The present study shows that IR combined with ATO has an increased therapeutic efficacy in LNCaP and PC-3 human prostate cancer cells. Specifically, the combined treatment induced autophagy and apoptosis in LNCaP cells, and mainly induced autophagy in PC-3 cells (Figs. 1, 2, 3). The above *in vitro* results are substantiated by *in vivo* experiments that employed a mouse models with xenograft tumors from PC-3 cells. The combined treatment suppressed tumor volume and tumor weight in nude mice compared with ATO or IR treatment alone (Fig. 6B). In addition, the combination therapy of IR and ATO resulted in a tumor growth inhibition of 74% (Table 1). Furthermore, the tumor tissues of LC3 and Atg5 expression were increased in the combined treatment compared with ATO or IR treatment alone (Fig. 6E).

Shen et al. indicated that ATO therapy is largely safe and few patients require cessation of treatment due to side effects [30]. The plasma level of arsenic in the clinical management of acute promyelocytic leukemia usually reaches 5.5–7.3 µM [30]. It has been reported that the peak concentration of plasma arsenic level was 10.6 µM after intraperitoneal administration of 0.2 mg (10 mg/kg) of ATO in mice [31]. The dosage used in our current study was 6 mg/kg ATO in mice represent a concentration of plasma arsenic level of 6.4 µM, which is clinically relevant. Furthermore, our results in xenograft mouse model found that there was no significant body weight loss in mice of combined treatment compared to control group (Fig. 6A), and therefore combined treatment procedure (6 mg/kg ATO three times per week for two weeks and a single dose of 6 Gy IR) was well tolerated.

The role of autophagy in cancer therapeutics is still controversial. Autophagy has been found to play a role as a cell survival mechanism that allows cells to clear damaged cytoplasmic proteins and organelles through lysosomal degradation and to survive metabolic stress [32]. On the other hand, autophagy has been found to contribute to type II programmed cell death in response to hypoxia, chemotherapeutic agents, viral infection, and toxins [33]. Our results suggest that the observed significant increase in autophagy can explain, at least in part, the cytotoxic effect of combined treatment because pre-treatment with 3-MA, an autophagy inhibitor, had a protective effect on combined treatment–induced cell death in LNCaP and PC-3 cells (Figs. 3, 5). Recent studies have found that the Akt/mTOR pathway plays a crucial role in the regulation of both apoptosis and autophagy [34]. Ser473 is important for the recognition and phosphorylation of Akt by PDK1 [35]. In addition, the antitumor effects of OSU-03012, a celecoxib derivative, were thought to be mainly mediated via the inhibition of PDK1 [36]. Disruption of the PI3K/Akt pathway, culminating in inhibition of Akt, has been found to be associated with autophagy induced by a variety of antineoplastic agents in cancer cells [34]. A combination of indol-3-carbinol and genistein synergistically induces apoptosis and autophagy in human colon cancer HT-29 cells by inhibiting Akt phosphorylation [37]. Friedrichs et al. indicated that polyunsaturated fatty acids can prevent the progression of prostate cancer cells to hormone independence by modifying signal transduction pathways such as the Akt/mTOR pathway [28]. An intact PI3K/Akt pathway is required for LNCaP cells to progress to hormone-refractory state, and Akt activity increases as the cells transform from hormone-dependent to hormone-independent [6]. The present study demonstrates that the expression of p-Akt, p-mTOR, p-p70S6K and p-PDK1 proteins decreased in cells treated with IR combined with ATO compared to treatment with ATO or IR alone (Fig. 4A).

Under oxidative stress, ROS, including free radicals, are generated at levels sufficient to induce oxidation and damage to DNA, lipids, proteins and other macromolecules [38], [39]. On occasion, autophagy and apoptosis both occur instantaneously after stress; at other times, only autophagy or apoptosis is observed [40]. ROS play a critical role in synergistic enhancement of apoptotic cell death by the combination treatment with IR and ATO in human cervical cancer cells [41]. It has been demonstrated that the induction of apoptosis by the treatment of cells with adenosine triggered caspase-3 activation, ROS formation and mitochondrial membrane potential (MMP) depletion in prostate cancer cell lines [42]. Recently, several studies have indicated that ROS may also be involved in the induction of autophagy. The production of ROS, which is a result of the loss of MMP, enhances the permeability of lysosomal membranes. This increased permeabikity leads to the release of lysosomal proteases, which contribute to mitochondrial membrane permeabilization and the lysosomal degradation mechanism of autophagic cell death [43]. H_2_O_2_ induces autophagy through interference with the beclin-1 and Akt/mTOR signaling pathways, and is regulated by the anti-apoptotic gene Bcl-2 in glioma U251 cells [44]. Phenethyl isothiocyanate triggers ROS to induce autophagy and apoptosis in human prostate cancer cells [45]. Consistent with these reports, we also found that combined treatment with ATO and IR significantly increases ROS generation and consequently induces autophagy in PC-3 cells (Figs. 2B, 2C). These results indicate that ROS is critically involved in combined treatment-induced cytotoxicity.

In conclusion, our results indicate that IR combined with ATO increases the therapeutic efficacy compared to individual treatments in LNCaP and PC-3 human prostate cancer cells. Specifically, combined treatment induced autophagy and apoptosis in LNCaP cells, and mainly induced autophagy in PC-3 cells. The combined treatment-induced cell death mainly occurred through inhibition of the Akt/mTOR signaling pathways, and ROS generation may be involved in the underlying mechanisms. In a nude mouse tumor xenograft model, the combination treatment possesses an anti-tumor growth effect. IR combined with ATO could is a novel therapeutic method that may be for the treatment of both androgen-dependent and -independent prostate cancer.
